# Response Surface Modeling and Optimization of Accelerated Solvent Extraction of Four Lignans from Fructus Schisandrae

**DOI:** 10.3390/molecules17043618

**Published:** 2012-03-23

**Authors:** Li-Chun Zhao, Ying He, Xin Deng, Geng-Liang Yang, Wei Li, Jian Liang, Qian-Li Tang

**Affiliations:** 1 Affiliated Ruikang Hospital of Guangxi University of Chinese Medicine, Nanning 530011, China; 2 College of Pharmacy, Hebei University, Baoding 071002, China; 3 College of Chinese Medicinal Materials, Jilin Agricultural University, Changchun 130118, China; 4 Department of Scientific Research, Guangxi University of Chinese Medicine, Nanning 530011, China

**Keywords:** accelerated solvent extraction, response surface methodology, lignans, Fructus Schisandrae

## Abstract

A new method based on accelerated solvent extraction (ASE) combined with response surface methodology (RSM) modeling and optimization has been developed for the extraction of four lignans in Fructus Schisandrae (the fruits of *Schisandra chinensis* Baill). The RSM method, based on a three level and three variable Box-Behnken design (BBD), was employed to obtain the optimal combination of extraction condition. In brief, the lignans schizandrin, schisandrol B, deoxyschizandrin and schisandrin B were optimally extracted with 87% ethanol as extraction solvent, extraction temperature of 160 °C, static extraction time of 10 min, extraction pressure of 1,500 psi, flush volume of 60% and one extraction cycle. The 3D response surface plot and the contour plot derived from the mathematical models were applied to determine the optimal conditions. Under the above conditions, the experimental value of four lignans was 14.72 mg/g, which is in close agreement with the value predicted by the model.

## 1. Introduction

Generally, the construction of mathematical models for prediction of target compound extraction and separation is an important tool in the field of natural product chemistry [1]. The models play a vital role in modeling and optimization of extraction processes leading to efficient and economical designs of actual operation [2,3]. As all we know, response surface methodology (RSM) is an effective modeling tool to solve linear and non-linear multivariate regression problems [4,5,6]. The RSM technique can simulate and optimize complex processes because it allows more efficient and easier arrangement and interpretation of experiments compared to other traditional method. For the extraction process, it is less laborious and time-consuming than other methods. In terms of these advantages, it is widely employed in optimizing the extraction of natural components including phenolics [6,7,8], chromones [4], saponins [3], and polysaccharides [9].

Recently, a great advance in extraction of bioactive compounds has been achieved using the accelerated solvent extraction (ASE) method [10,11,12], which represents an exceptionally effective extraction technique compared to alternative methods such as Soxhlet extraction (SE), heat-reflux extraction (HRE) and ultrasound-assisted extraction (UAE). Accelerated solvent extraction (ASE) is a fully automated technique that uses common solvents to rapidly extract solid and semisolid samples. ASE operates at temperature above the normal boiling point of most solvents, using pressure to keep the solvents in liquid form during the extraction process. This extraction technique leads to the possibility of automation, low solvent volume and reduced extraction times [13,14]. In addition, the kinetic processes are accelerated for target components to desorb from the matrix at high pressure [15,16,17,18].

**Figure 1:**
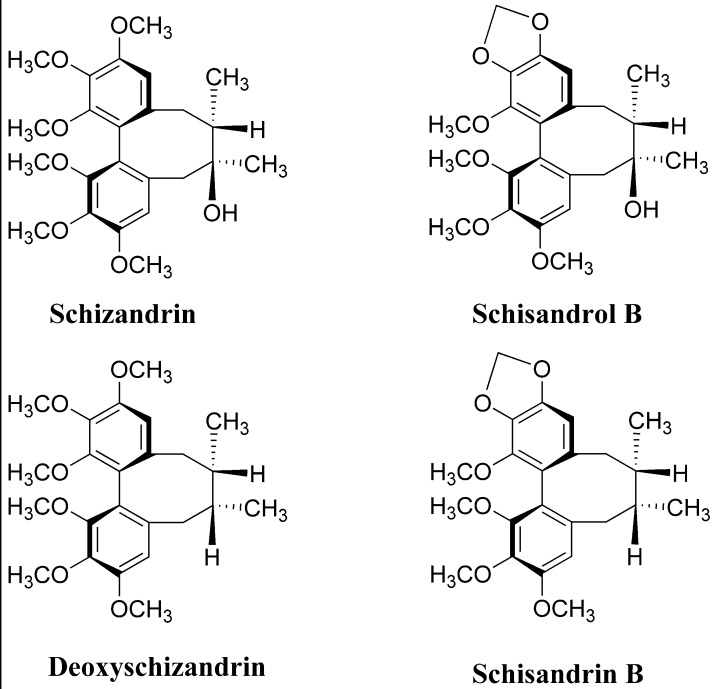
Chemical structures of schizandrin, schisandrol B, deoxyschizandrin and schisandrin B.

Many reports dating from the early 19th century have indicated the therapeutic properties of Fructus Schisandrae (the fruits of *Schisandra chinensis* Baill) for liver diseases, including prevention of liver damage, stimulation of liver regeneration and inhibition of hepatocarcinogenesis [19,20]. Lignans, the major active principle from *S*. *chinensis*, have shown satisfactory evidence for a clinically significant activity in chronic liver injuries [21,22]. The chemical structures of lignans in this plant have been extensively investigated in many published papers, and so far more than 30 lignans have been isolated and identified [23]. Among them, the major ones were the four lignans schizandrin, schisandrol B, deoxyschizandrin and schisandrin B (Figure 1). Although the ASE method has proved to be effective for the extraction of natural compounds from medicinal plants, no study exist on the simultaneous extraction of four lignans from *S*. *chinensis* using RSM modeling and optimization.

This study aimed to apply response surface methodology (RSM) to model and optimize ASE technique for the extraction of four lignans from Fructus Schisandrae. Several important factors, such as extraction solvent, extraction time and extraction temperature, were systemically analyzed using a Box-Behnken design. To the best of our knowledge, this is the first report on combination of RSM and ASE for extraction of four lignans from Fructus Schisandrae.

## 2. Results and Discussion

### 2.1. Predicted Model and Statistical Analysis

Preliminary studies were performed in order to determine the required range of ethanol concentrations (*X*_1_, 50–100%), extraction time (*X*_2_, 5–12 min) and extraction temperature (*X*_3_, 80–180 °C). The whole design consisted of 15 experimental points as listed in Table 1, and three replicates (run 13–15) at the center of the design were used for estimating a pure error sum of squares. The experiments were performed in triplicate at all design points in randomized order.

**Table 1:** Factors and levels for RSM, and Box-Behnken design with the independent variables.

Run	Coded and Uncoded Variables Levels			Yield of Lignans (mg/g)	
	*X*_1_/ethanol (%)	*X*_2_/time (min)	*X*_3_/temperature (°C)	Actual Values	Predicted Values
1	−1 (50)	−1 (5)	0 (130)	9.96 ± 0.32	9.94
2	1 (100)	−1 (5)	0 (130)	11.34 ± 0.25	11.15
3	−1 (50)	1 (12)	0 (130)	12.65 ± 0.34	12.85
4	1 (100)	1 (12)	0 (130)	13.99 ± 0.45	14.01
5	−1 (50)	0 (8.5)	−1 (80)	11.80 ± 0.26	11.69
6	1 (100)	0 (8.5)	−1 (80)	12.77 ± 0.23	12.83
7	−1 (50)	0 (8.5)	1 (180)	12.88 ± 0.33	12.81
8	1 (100)	0 (8.5)	1 (180)	13.91 ± 0.29	14.03
9	0 (75)	−1 (5)	−1 (80)	9.90 ± 0.22	10.03
10	0 (75)	1 (12)	−1 (80)	12.99 ± 0.21	12.91
11	0 (75)	−1 (5)	1 (180)	11.10 ± 0.22	11.19
12	0 (75)	1 (12)	1 (180)	14.21 ± 0.31	14.08
13	0 (75)	0 (8.5)	0 (130)	14.12 ± 0.28	14.00
14	0 (75)	0 (8.5)	0 (130)	13.89 ± 0.23	14.00
15	0 (75)	0 (8.5)	0 (130)	13.99 ± 0.27	14.00

The design matrix and the corresponding results of RSM experiments to determine the effects of the three independent variables including ethanol concentrations, extraction time and extraction temperature were shown in Table 2. Through multiple regression analysis on the experimental data, predicted response *Y* for the yield of lignans could be expressed by the following second-order polynomial equation in term of coded values:

*Y *= 14 + 0.59*X*_1_ + 1.44*X*_2_ + 0.58*X*_3_ − 0.011*X*_1_*X*_2_ + 0.018*X*_1_*X*_3_ + 3.500E − 003*X*_2_*X*_3_ − 0.61*X^2^_1_* − 1.40*X^2^_2_* − 0.55*X^2^_3_*

where *Y* is the yield of four lignans (mg/g), and *X*_1_, *X*_2_ and *X*_3_ are the coded variables for ethanol concentration, extraction time and extraction temperature, respectively. Statistical testing of the model was performed in the form of analysis of variance (ANOVA). Then ANOVA for the fitted quadratic polynomial model of extraction of four lignans were shown in Table 2. The quadratic regression model showed the value of determination coefficient (*R^2^*) of 0.9939 with no significant lack of fit at *p *> 0.05, which means that the calculated model was able to explain 99.39% of the results. The results indicated that the model used to fit response variable was significant (*p *< 0.0001) and adequate to represent the relationship between the response and the independent variables [24]. The significance of the model was also judged by *F*-test, which suggested that model had a very high model *F*-value (*F* = 125.80). *R*^2^_a__dj_ (adjusted determination coefficient) is the correlation measure for testing the goodness-of-fit of the regression equation [25]. The *R*^2^_adj_ value of this model is 0.9860, which indicated only 1.40% of the total variations were not explained by model. Meanwhile, a relatively lower value of coefficient of variation (CV = 1.36) showed a better precision and reliability of the experiments carried out [4,26].

**Table 2:** Analysis of variance for the fitted quadratic polynomial model of extraction of lignans.

Source	SS	DF	MS	*F*-value	*p*-value	
Model	34.27	9	3.81	125.80	<0.0001	significant
Residual	0.21	7	0.030			
Lack of fit	0.16	3	0.054	4.38	0.0938	insignificant
Experimental error	0.049	4	0.012			

Notes: SS, sum of squares; DF, degree of freedom; MS, mean square.

The significance of each coefficient was also determined using *F*-value and *p*-value. The results are given in Table 3. It could be seen that the lignans extraction yield was affected significantly by all three extraction parameters (*X*_1_, *X*_2_, and *X*_3_, *p *< 0.001). In addition, it was evident that all the quadratic parameters (*X^2^_1_*, *X^2^_2_*, *X^2^_3_*) were significant at the level of *p *< 0.05 or *p *< 0.0001, whereas the interaction quadratic parameters (*X*_1_*X*_2_,*X*_1_*X*_3_ and *X*_2_*X*_3_) were insignificant (*p *> 0.1).

**Table 3:** Estimated regression model of relationship between response variables (yield of four lignans) and independent variables (*X*_1_, *X*_2_, *X*_3_).

Variables	DF	SS	MS	*F*-values	*p*-value
*X*_1_	1	2.79	2.79	92.25	<0.0001
*X*_2_	1	16.65	16.65	550.04	<0.0001
*X*_3_	1	2.70	2.70	89.15	<0.0001
*X^2^_1_*	1	1.59	1.59	52.41	0.0002
*X^2^_2_*	1	8.28	8.28	273.56	<0.0001
*X^2^_3_*	1	1.27	1.27	41.97	0.0003
*X*_1_ *X*_2_	1	4.41E-004	4.41E-004	0.015	0.9073
*X*_1_ *X*_3_	1	1.23E-003	1.23E-003	0.040	0.8463
*X*_2_ *X*_3_	1	4.90E-005	4.90E-005	1.62E-003	0.9690

Notes: SS, sum of squares; DF, degree of freedom; MS, mean square.

### 2.2. Optimization of Extraction Parameters of Lignans

**Figure 2:**
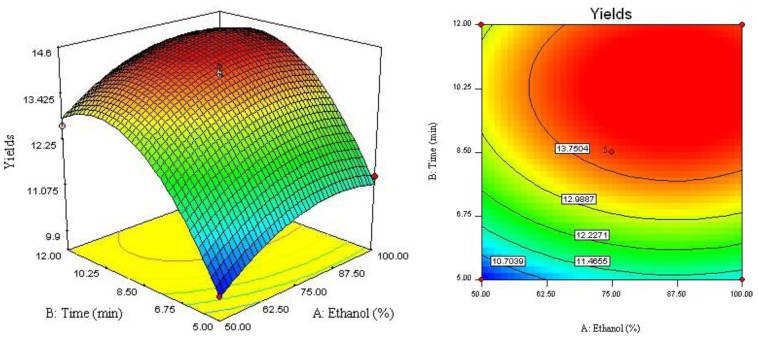
Response surface plot and contour plot of ethanol concentration and extraction time.

**Figure 3:**
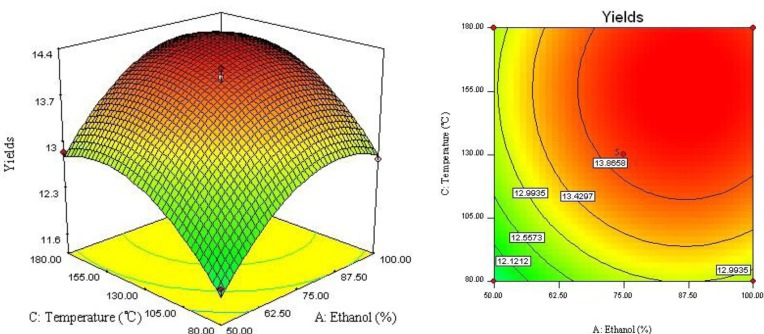
Response surface plot and contour plot of ethanol concentration and extraction temperature.

**Figure 4:**
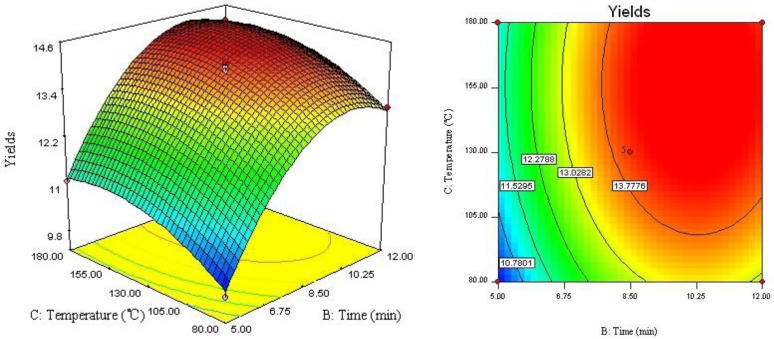
Response surface plot and contour plot of extraction temperature and extraction time.

The relationship between independent and dependent variables was graphically represented by 3D response surface and 2D contour plots generated by the model (Figure 2, Figure 3, Figure 4). Different shapes of the contour plots indicated different interactions between the variables, an elliptical contour plot indicated the interactions between the variables were significant while a circular contour plot means otherwise.

Figure 2 showed the interaction between ethanol concentration (*X*_1_) and extraction time (*X*_2_) on the yield of four lignans. Increase of ethanol concentration from 50 to 87% improve the extraction yield. However, when the ethanol concentration over 87%, there was a gradual decline in the response, and extraction time over 10 min did not show any obvious effect on extraction yield. The possible explanation could be that, increasing extraction time may accelerate chemical degradation of bioactive compound in extraction process, which resulted in the lower extraction yield [11]. Figure 3 described the effect of ethanol concentration (*X*_1_) and extraction temperature (*X*_3_) on the yield of four lignans. It may be also observed that when extraction time (*X*_2_) was fixed at 0 level, extraction temperature (*X*_3_) displayed a quadratic effect on the response yield. Varying ethanol concentration from 50 to 90% with increase of extraction temperature from 80 to 160 °C, the target compounds’ extraction yield was increasing with increase of ethanol concentration. As shown in Figure 4 and Table 3, when ethanol concentration (*X*_1_) was fixed at 0 level, the interaction of extraction time (*X*_2_) and extraction temperature (*X*_3_) had also insignificant effect on the extraction yields. It depicted that the highest extraction yield could be achieved when using near 160 °C of extraction temperature and 10 min of extraction time.

### 2.3. Selection of Extraction Cycles and Flush Volume

The extraction cycles of ASE was determined by performing consecutive accelerated solvent extractions three cycles on the same sample. According to pervious studies and present investigation, single extraction cycle was sufficient to completely extract most of target components [11,27]. In order to save time and energy, one cycle extraction was employed in this investigation (data not shown).

In addition, different flush volume (20, 40, 60, and 80%) was evaluated on whether it affects extraction efficiencies of the target compounds. The results indicated that flush volume was not the main influencing factor (data not shown). Therefore, the flush volume was set at its default values of 60%. Given that extraction pressure was fixed in ASE 300 system (1,500 psi) [27], we did not evaluate the effect of extraction pressure here.

### 2.4. Verification of Predictive Model

In this study, the aim of optimization was to find the conditions which gave the maximum extraction yield of four lignans in Fructus Schisandrae. The software generated the optimum ethanol concentration, extraction time and extraction temperature was 87.09%, 10.30 min and 156.94 °C, respectively. The software predicted the extraction yield of total lignans was 14.67 mg/g. As shown in Table 4, three parallel experiments were carried out under the optimal conditions. Comparing with the value predicted by Design Expert 7.1.6, the results showed that the actual value of 14.72 mg/g was very close to the actual results. In addition, the extraction yield of crude extract was 11.21%, and all target compounds were extracted absolutely via HPLC analysis of sample residues. This indicated that the optimization achieved in the present study was reliable.

**Table 4:** Optimum conditions and the predicted and experimental value of response at the optimum conditions.

	Ethanol (%)	Extraction time (min)	Temperature (°C)	Yield of Lignans (mg/g)
**Optimum conditions (predicted)**	87.09	10.30	156.94	14.67
**Modified conditions (actual)**	87.00	10.00	160.00	14.72 ± 0.77

### 2.5. Comparison with Conventional Extractions

Prior to the present investigation, heat-reflux extraction was used for sample preparation of lignans in Fructus Schisandrae [28]. Hence, it is necessary to compare ASE with HRE on extraction yield of total lignans. The results indicated the lignans’ yield of HRE (13.55 mg/g) was slightly lower than that of ASE (14.72 mg/g). Moreover, ASE took only one twelfth of the time it took for HRE (2 h). Therefore, ASE can save lots of time comparing to HRE and provide higher yield of lignans than HRE

## 3. Experimental

### 3.1. Plant Material

Fructus Schisandrae, the wild fruits of *Schisandra chinensis* Baill, were obtained from Changchun Huarui Ginseng Co., Ltd., and identified by Wei Li (College of Chinese Medicinal Materials, Jilin Agricultural University). The cut pieces were ground to obtain a relatively homogenous drug powder (80–100 mm). The powder was dried at 60 °C until constant weight and was well blended before use.

### 3.2. Chemicals and Reagents

Methanol for chromatographic grade was purchased from Fisher Chemicals (Miami, FL, USA). Other chemicals, such as ethanol, *etc*. were all of analytical grade from Beijing Chemical Factory. Water was purified using a Milli-Q water purification system (Milipore, Boston, MA, USA). Standards of schizandrin, schisandrol B, deoxyschizandrin and schisandrin B were obtained from the National Institute for the Control of Pharmaceutical and Biological Products of China (Beijing, China).

### 3.3. Accelerated Solvent Extraction

ASE was performed with a Dionex ASE 300^TM^ instrument equipped with a solvent controller (Dionex, Sunnyvale, CA, USA). The system consists of a high pressure pneumatic solvent pump capable of 1,500 psi at elevated flow rate; an extraction solvent pressurized bottle; a carousel for 12 extraction cells of 100 mL; a carousel for 250 mL collection vials; a microprocessor for storing and editing parameters such as temperature, time and pressure; infra-red (IR) sensors to detect the fluid arrival into the collection vial and monitor fluid levels during extract collection. In present investigation, 100 mL of extraction cells was used and filled with 5.0 g of sample powders.

After the extraction procedure, the filtered solutions were concentrated to dryness in vacuum at 45 °C. The obtained dry extracts were diluted in 25 mL ethanol and the supernatant was filtered through a 0.45 μm nylon membrane and then injected into HPLC for analysis. Three replicate injections were analyzed to determine the extraction yields of lignans with the mean peak area. To evaluate the repeatability of the extraction procedure, a series of three replicated extractions were performed. The results obtained for all target compounds revealed that the R.S.D. was lower than 3.7%.

### 3.4. Conventional Extraction

Heat-reflux extraction (HRE) as one of conventional extractions was performed according to the one published report [28]. In brief, exhaustive extraction with ethanol was performed on 2.0 g drug powder, placed in an extraction bag filter, and impregnated with ethanol. Extraction was performed for about 2 h with 50 mL methanol.

### 3.5. HPLC Analysis of Four Lignans

**Figure 5:**
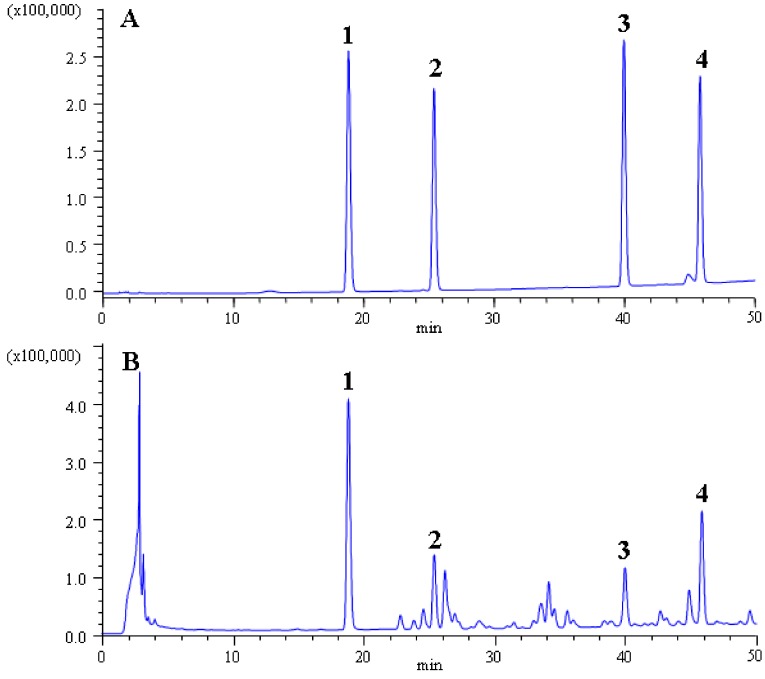
HPLC chromatograms of standard solution of four lignans (**A**); extracts of *S. Chinensis* (**B**); schizandrin (1), schisandrol B (2), deoxyschizandrin (3) and schisandrin B (4).

In the present work, four lignans in Fructus Schisandrae were quantified simultaneously by high performance liquid chromatography coupled with UV detection (HPLC-UV; Figure 5). The HPLC analyses were performed with a HPLC instrument (Agilent 1100, Agilent Technologies, Los Angeles, CA, USA) equipped with a quaternary solvent delivery system, a column oven and UV detector. Separation was achieved on Hypersil ODS2 column (4.6 mm × 250 mm, 5 μm) from Dalian Elite Analytical Instruments Co., Ltd. (Dalian, China). The column temperature was set at 25 °C and detection wavelength was set at 254 nm. The mobile phase was consisted of water (A) and methanol (B) with flow rate of 1.0 mL/min. The gradient elution was programmed as follows: 0–5min, 50% B; 5–45 min, 50–80% B; 45–55 min, 80% B.

### 3.6. Experimental Design and Statistical Analyses

A single-factor-test was employed to determine the preliminary range of the extraction variables including *X*_1_ (ethanol concentration), *X*_2_ (extraction time) and *X*_3_ (extraction temperature). Then, a three-level-three-factor Box-Behnken factorial design (BBD) (Design Expert software, Trial Version 7.1.6, Stat-Ease Inc., Minneapolis, MN, USA) was applied to determine the best combination of extraction variables for the yields of four lignans in Fructus Schisandrae.

Experimental data were fitted to a quadratic polynomial model and regression coefficient obtained. The non-linear computer-generated quadratic model used in the response surface was as follows:


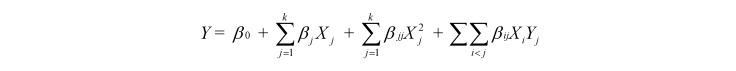


where *Y* is the estimated response, *β_0_*, *β_j_*, *β_jj_* and *β_ij_* are the regression coefficients for intercept, linearity, square and interaction, respectively, while *X_i_*, *X_j_* are the independent coded variables. Design Expert software was used to estimate the response of each set of experimental design and optimized conditions. The fitness of the polynomial model equation was expressed by the coefficient *R*^2^. *F*-test and *p*-value were used to check the significance of the regression coefficient. Data were expressed as the means (SEM) of three replicated determinations.

## 4. Conclusions

In this study, RSM was used to model and optimize ASE method for extraction of four lignans in Fructus Schisandrae. It was effective for estimating the effect of three main independent variables (extraction solvent, time, and temperature) by using the contour and surface plots in RSM. In addition, a second-order polynomial model was employed to optimize lignans extraction from *S. chinensis* by ASE technology. The optimal extraction conditions for the lignans were as follows: 87% ethanol of extraction solvent, 160 °C of extraction temperature, 10 min of extraction time, 1,500 psi of extraction pressure, flush volume of 60% and one extraction cycle. Under the optimum conditions, the experimental extraction yields of four lignans agreed closely with the predicted yield of 14.72 mg/g. In brief, the present study provided a new and efficient method for the extraction of lignans from *S. chinensis*.
